# Commentary: The *m*-AAA Protease Associated with Neurodegeneration Limits MCU Activity in Mitochondria

**DOI:** 10.3389/fphys.2016.00583

**Published:** 2016-11-25

**Authors:** Paolo Bernardi, Michael Forte

**Affiliations:** ^1^Department of Biomedical Sciences, University of PadovaPadova, Italy; ^2^Vollum Institute, Oregon Health and Science UniversityPortland, OR, USA

**Keywords:** mitochondria, proteases, calcium uniporter, permeability transition pore, neurodegenerative diseases

SPG7 and AFG3L are *m*-AAA proteases that form homo-oligomeric complexes and perform essential functions in mitochondrial quality control (Quirós et al., 2015). Mutations of the SPG7 and AFG3L genes cause an autosomal recessive form of hereditary spastic paraplegia (Casari et al., 1998) and spinocerebellar ataxia (Atorino et al., 2003; Di Bella et al., 2010), respectively, as well as other neurological syndromes (Pierson et al., 2011). Mitochondrial dysfunction plays a fundamental role in disease onset, as suggested by decreased activity of respiratory complex I and increased sensitivity to reactive oxygen species (Atorino et al., 2003). König et al. now show that *m*-AAA proteases assist in the correct formation of the mitochondrial Ca^2+^ uniporter (MCU) complex by regulating the levels of its essential subunit EMRE (König et al., 2016). The study provides a potential mechanistic link between *m*-AAA protease dysfunction, deregulation of Ca^2+^ homeostasis and opening of the permeability transition pore (PTP), a high conductance channel that requires matrix Ca^2+^ and is stimulated by oxidative stress (Bernardi et al., 2015). The suggestion that SPG7 is part of the PTP (Shanmughapriya et al., 2015) is seriously questioned by the findings of the article covered in this editorial, supporting the view outlined in a previous commentary (Bernardi and Forte, 2015).

In energized mitochondria the Ca^2+^ electrochemical gradient drives Ca^2+^ uptake through the MCU (Baughman et al., 2011; De Stefani et al., 2011). MCU function is affected by the regulatory subunits MICU1 (Perocchi et al., 2010), MICU2 (Plovanich et al., 2013), the MCU inhibitory paralog MCUb (Raffaello et al., 2013) and EMRE (Sancak et al., 2013), a sensor for matrix Ca^2+^ and a critical gatekeeper for the MCU (Vais et al., 2016). In the absence of EMRE (which faces the matrix) MCU currents are no longer inhibited by matrix Ca^2+^ (which would normally limit its accumulation) resulting in enhanced mitochondrial Ca^2+^ uptake and elevation of matrix [Ca^2+^] (Vais et al., 2016). It was already known (and somewhat puzzling) that EMRE-dependent regulation of MCU channel activity requires both MICU1 and MICU2, which are localized on the opposite side of the inner membrane and face the intermembrane space (Vais et al., 2016). The results of König et al. bear on this issue with the novel finding that EMRE is a substrate of *m*-AAA proteases in the intermembrane space before its import and incorporation into the MICU1-MICU2 complex during the dynamic processes that ultimately generate the complete, regulated MCU complex. In the absence of adequate *m*-AAA protease activity, the excess EMRE associates with the MCU in a complex that lacks MICU1 and MICU2 (König et al., 2016). This unprocessed, unregulated MCU-EMRE complex would not be inhibited by matrix Ca^2+^ and thus mediate excessive mitochondrial Ca^2+^ uptake, leading to opening of the PTP and to all its detrimental consequences linked to deregulation of Ca^2+^ homeostasis and ATP depletion (Bernardi et al., 2015). Consistent with this picture, mitochondria from HeLa cells and from mice lacking SPG7 and/or AFG3L displayed increased sensitivity to Ca^2+^-induced PTP opening (König et al., 2016), as measured with the Ca^2+^ retention capacity (CRC) assay, i.e., the amount of Ca^2+^ needed to trigger pore opening. This is the opposite of what was reported by Shanmughapriya et al. who carried out a phenotypic screen of the CRC in permeabilized cells after inactivation of a variety of genes. According to this report, SPG7 downregulation *desensitized* the PTP by rendering it less sensitive to Ca^2+^ (Shanmughapriya et al., 2015). In contrast, König et al. find that depletion of SPG7 *facilitated* PTP opening under all conditions tested (König et al., 2016). Irrespective of the basis for this discrepancy, which might depend on complementation by homo-oligomeric AFG3L2 and will need further investigation, the predictions of the two sets of opposing findings can be matched against the known hallmarks of diseases linked to mutations of *m*-AAA proteases.

Mitochondrial dysfunction is of pathogenic relevance to neurodegenerative disorders due to mutations of *m*-AAA proteases. This is clearly indicated by the presence of abnormal, often swollen mitochondria (Casari et al., 1998; Atorino et al., 2003; Ferreirinha et al., 2004; Di Bella et al., 2010; Almajan et al., 2012), which is the expected outcome of PTP opening (Bernardi et al., 2015). The second element is derangement of Ca^2+^ homeostasis since both downregulation of the glutamate receptor, which mediates Ca^2+^-dependent PTP opening (Schinder et al., 1996), and decrease of cytosolic [Ca^2+^] suppressed ataxia in an AFG3L2-deficent mouse (Maltecca et al., 2015). Thus, the pathological alterations are best explained by PTP sensitization (König et al., 2016) rather than by PTP inhibition (Shanmughapriya et al., 2015). It should be noted that decreased activity of respiratory complex I and increased reactive oxygen species (Atorino et al., 2003) can cause sensitization to PTP opening even if the increase of mitochondrial [Ca^2+^] is not large (e.g., Figure 6C). *m*-AAA proteases possess only 2 hydrophobic regions (Casari et al., 1998; Banfi et al., 1999) and are therefore unlikely to form high-conductance channels. We think that this missing piece of evidence, which is essential to substantiate the claim that SPG7 is a constituent of the PTP (Shanmughapriya et al., 2015), should be provided before this hypothesis can be considered further. For the time being we believe that an indirect, PTP inducing effect of defective *m*-AAA proteases is the most plausible explanation for the pathogenesis of neurological diseases due to mutations in their genes. Formation of deregulated MCU complexes provides a provocative but testable mechanism for the increased probability of PTP opening.

## Author contributions

PB and MF wrote the commentary.

### Conflict of interest statement

The authors declare that the research was conducted in the absence of any commercial or financial relationships that could be construed as a potential conflict of interest. The reviewer JH and handling Editor declared their shared affiliation, and the handling Editor states that the process nevertheless met the standards of a fair and objective review.
